# Microbial Diversity Analysis of Fermented Mung Beans (Lu-Doh-Huang) by Using Pyrosequencing and Culture Methods

**DOI:** 10.1371/journal.pone.0063816

**Published:** 2013-05-20

**Authors:** Shiou-Huei Chao, Hui-Yu Huang, Chuan-Hsiung Chang, Chih-Hsien Yang, Wei-Shen Cheng, Ya-Huei Kang, Koichi Watanabe, Ying-Chieh Tsai

**Affiliations:** 1 Institute of Biochemistry and Molecular Biology, National Yang-Ming University, Taipei, Taiwan; 2 Department of Food Science, Nutrition and Nutraceutical Biotechnology, Shih Chien University, Taipei, Taiwan; 3 Center for Systems and Synthetic Biology, National Yang-Ming University, Taipei, Taiwan; 4 Institute of Biomedical Informatics, National Yang-Ming University, Taipei, Taiwan; 5 Yakult Central Institute for Microbiological Research, Tokyo, Japan; Charité-University Medicine Berlin, Germany

## Abstract

In Taiwanese alternative medicine Lu-doh-huang (also called *Pracparatum mungo*), mung beans are mixed with various herbal medicines and undergo a 4-stage process of anaerobic fermentation. Here we used high-throughput sequencing of the 16S rRNA gene to profile the bacterial community structure of Lu-doh-huang samples. Pyrosequencing of samples obtained at 7 points during fermentation revealed 9 phyla, 264 genera, and 586 species of bacteria. While mung beans were inside bamboo sections (stages 1 and 2 of the fermentation process), family *Lactobacillaceae* and genus *Lactobacillus* emerged in highest abundance; *Lactobacillus plantarum* was broadly distributed among these samples. During stage 3, the bacterial distribution shifted to family *Porphyromonadaceae*, and *Butyricimonas virosa* became the predominant microbial component. Thereafter, bacterial counts decreased dramatically, and organisms were too few to be detected during stage 4. In addition, the microbial compositions of the liquids used for soaking bamboo sections were dramatically different: *Exiguobacterium mexicanum* predominated in the fermented soybean solution whereas *B. virosa* was predominant in running spring water. Furthermore, our results from pyrosequencing paralleled those we obtained by using the traditional culture method, which targets lactic acid bacteria. In conclusion, the microbial communities during Lu-doh-huang fermentation were markedly diverse, and pyrosequencing revealed a complete picture of the microbial consortium.

## Introduction

Lu-doh-huang (also called *Pracparatum mungo*), which is based on fermented mung beans (Vigna *radiata), is a Taiwanese alternative medicine. In this setting, Lu-doh-huang is used for its antipyretic and diuretic effects and for detoxification, reduction of swelling, and gut decontamination as empiric treatment. In addition, Lu-doh-huang induces apoptosis of Hep3B hepatoma cells [1], reduces inflammation in lipopolysaccharide-induced RAW264.7 cells [2], and has antioxidative properties [2]–[4]. In animal models, Lu-doh-huang exerts a protective effect on CCl_4_-induced hepatotoxicity in rats [5]; induces the activities of NADPH-CYP reductase, glutathione S-transferase, superoxide dismutase, glutathione peroxidase, and catalase in Balb/c mice [2], [3]; significantly decreases plasma glucose, total cholesterol, and triglycerides [3]; and inhibits tyrosinase activity in MDCK and A375 cells [3].*

**Figure 1:**
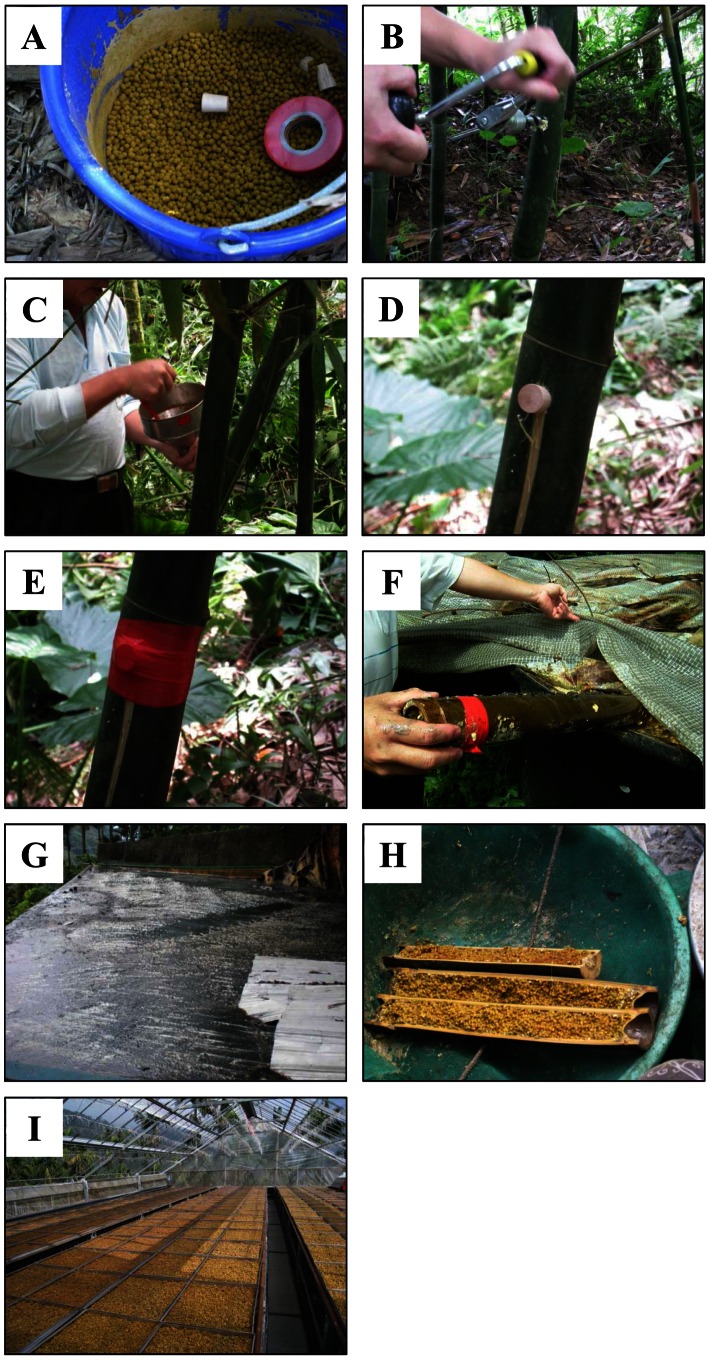
Lu-doh-huang manufacture processes. Images (a) through (e) represent stage 1 in Figure 2. Image (f) represents stage 2, (g) illustrates stage 3, and (h) and (i) correspond to stage 4.

**Figure 2:**
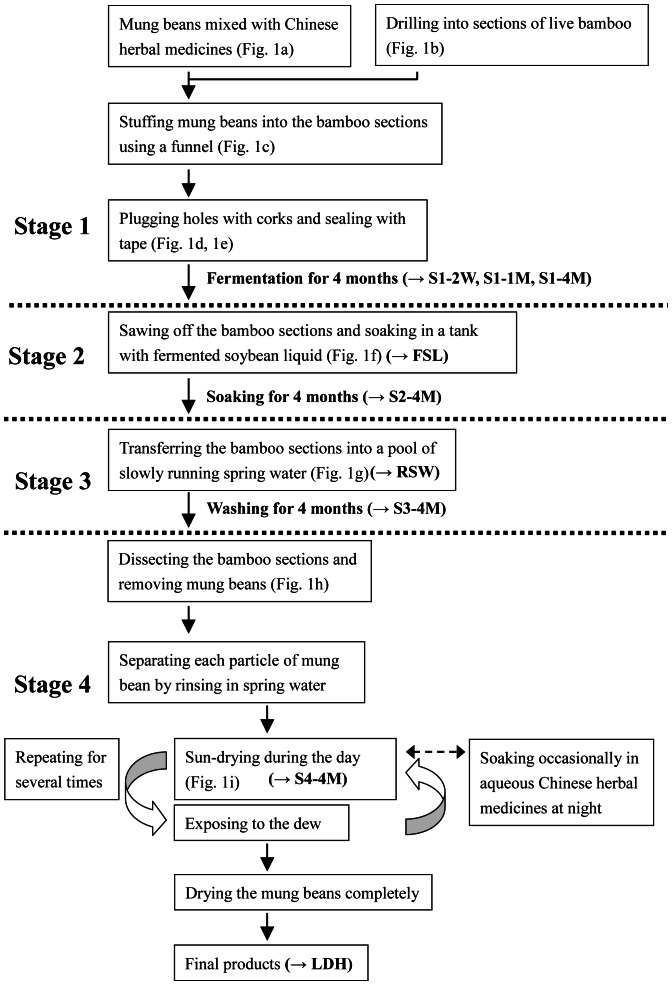
Flowchart of the Lu-doh-huang manufacturing process. Sampling points (in bold) are shown in brackets.

The manufacturing process of Lu-doh-huang is divided into 4 stages, and each stage lasts for 4 months (Fig. 1 and 2). Stage 1 runs from about the beginning of September to the end of the following January. During this stage, mung beans are mixed with various Chinese herbal medicines, including powdered *Ganoderma lucidum*, *Taiwanofungus camphoratus*, *Coptis chinensis* Franch., *Houttuynia cordata* Thunb., and *Glycyrrhiza uralensis* Fisch. Small- diameter holes are drilled into sections of live bamboo. The mixture of mung beans then is stuffed through these holes in the bamboo by using a funnel; once the section is filled, these holes are corked and sealed tightly with tape. During stage 2, the bean-filled bamboo sections are sawn open and soaked in a spontaneously fermented soybean solution; these bamboo sections are transferred to a pool of slowly running spring water to soak during stage 3. At stage 4, the bamboo sections are cleaved into two so that the mung beans can be removed, rinsed in spring water, and sun-dried during the day. At night, the mung beans are soaked in an aqueous mixture of Chinese herbal medicines or put outside to expose them to the dew. These steps are repeated several times until the mung beans are dried completely to become the final product. Although a wide variety of fermented soybean products are used worldwide– including chungkokjang (Korea), kinema (India), miso (Japan), natto (Japan), shoyu (China and Japan), and tempeh (Indonesia)–none results from a fermentation process similar to that for Lu-doh-huang. Whereas the medical applications of Lu-doh-huang have been explored in several studies, the composition of the bacterial communities during its manufacture remains unknown. In the current study, we used pyrosequencing to assess the microbial diversity of Lu-doh-huang during its production by fermentation and applied the traditional culture method to identify the predominant lactic acid bacteria (LAB) during Lu-doh-huang fermentation.

## Materials and Methods

### Sample Collection and pH Analysis

Throughout the 4-stage process, we collected samples of the mung beans inside bamboo sections and the liquids in which the bamboo sections were soaked. At least 3 samples each of beans and liquid were collected at each of 7 points during the manufacturing process; like samples were pooled for further analysis. During stage 1, mung beans were fermented in sections of live bamboo; samples were collected after 2 weeks (S1–2W), 1 month (S1–1M), and 4 months (S1–4M) of fermentation. During stage 2, the bamboo sections containing mung beans were soaked in fermented soybean liquid (FSL) for 4 months (the S2–4M sample); during stage 3, the bamboo sections were soaked in slowly running spring water (RSW) for 4 months (the S3–4M sample). During stage 4, the mung beans were removed from the bamboo sections and then repeatedly sun-dried during the day and exposed to dew (or Chinese herbal medicine) during the night for 4 months (S4–4M). The fermented mung beans then were dried to yield the final product, Lu-doh-huang (LDH). Samples of the unfermented material (UFM) and the FSL and RSW in which the filled bamboo sections were soaked were collected also. The samples were stored on ice during transport to the laboratory for analysis within 7 days of sampling. The pH of the samples was measured (model PHB-9901, DO Microelectronic pH-Vision meter, Ai-On Industrial Corporation, Taipei, Taiwan).

### PCR Amplification for Pyrosequencing

The V1–V2 hypervariable regions of the 16S rRNA genes of bacteria present at each sampling point were amplified individually by using nested PCR as previously described [6]. For the first round of amplification, primers 27f (5′-AGRGTTTGATYMTGGCTCAG-3′) and 338r (5′- TGCTGCCTCCCGTAGGAGT-3′) were used. Each reaction (volume, 25 µL) contained 10 ng of extracted DNA, 10 mM Tris-HCl (pH 8.3), 50 mM KCl, 1.5 mM MgCl_2_, 200 µM deoxynucleoside triphosphate (dNTP) mixture, 5 pmol of each primer, and 0.625 U ExTaq HS (Takara Bio, Shiga, Japan). Thermocycling conditions were: 1 cycle of 98°C for 2.5 min; 15 cycles of 98°C for 15 s, 50°C for 30 s, and 72°C for 20 s; and a final cycle of 72°C for 5 min. The second round of PCR was performed by using the primers Pyro-27f (5′-CCATCTCATCCCTGCGTGTCTCCGACtcagN_10_ *AGRGTTTGATYMTGGCTCAG*-3′) and Pyro-338r (5′-CCTATCCCCTGTGTGCCTTGGCAGTCtcag*TGCTGCCTCCCGTAGGAGT*-3′). The underlined sequences are dictated by the requirements of the 454 Sequencing System (Roche Diagnostics, Branford, CT, USA); the nucleotides in lowercase letters are sequence keys used for amplicon sequencing; italicized sequences are those for universal primers for bacteria; and N_10_ designates the 10-base multiplex identifiers (Roche Diagnostics) used to label each PCR product. The second round of PCR amplification was performed in a 50-µL volume containing 0.5 µL of the product of the first-round of PCR, 10 pmol of each primer, and 1.25 U Ex Taq HS (TaKaRa Bio., Shiga, Japan); all other components were the same as for the first round of PCR. Cycling conditions for the second round of PCR were: 1 cycle of 98°C for 2.5 min; 15 cycles at 98°C for 15 s, 54°C for 30 s, and 72°C for 20 s; and a final cycle of 72°C for 5 min.

The quantity and quality of amplicons were determined by using the Quant-iT PicoGreen dsDNA Assay Kit (Molecular Probes, OR. USA) and Agilent DNA 1000 chip, respectively, on a BioAnalyzer 2100 (Agilent Technologies, CA, USA). Each amplicon was purified separately by using Agencourt AMPure XP beads (Beckman Coulter, Brea, CA, USA) according to the manufacturer’s protocol. Purified amplicons were quantified, diluted to 1×10^9^ molecules/µL, and underwent a second purification step by using Agencourt AMPure XP beads (Beckman Coulter). Samples with different sample-specific barcode sequences were quantified and diluted to 1×10^8^ molecules/µL each and pooled proportionally. The quality of the amplicon pool was assessed by using a High-Sensitivity DNA chip (Agilent Technologies) and subsequently subjected to emulsion PCR by using a GS emPCR Lib-L Kit (Roche Diagnostics). Pyrosequencing was performed on a GS Junior System (Roche Diagnostics) by using Titanium chemistry (Roche Diagnostics) according to the manufacturer’s instructions.

### Analysis of Pyrosequencing Data

Raw Fasta data were trimmed by using FASTX-Toolkit [7] (Q>25); reads were recognized as derived from specific samples by their unique multiplex identifier. After removing the multiplex identifier and primer sequences for each read, only reads longer than 310 nucleotides were chosen for taxonomic assignment by using BLASTn [8]. High-quality trimmed reads were used as BLASTn queries against the 9701 full-length (≥1200 nucleotides) high-quality 16S rRNA sequences from isolated typed strains (September 2012, RDP release 10, update 30) [9]. In BLASTn results, only the best hit queried by each read was considered. Only reads with more than 97% sequence identity were assigned to the corresponding species. Aligned sequences were clustered into operational taxonomic units (OTU) defined at 3% sequence distance by using the complete-linkage clustering tool in RDP [9]. The Shannon–Weaver index [10], Chao1 index, and evenness and rarefaction analyses also were completed by using the web-based services of RDP.

The relative abundance of sequence reads at the bacterial genus level for seven samples were studied by principal component analysis (PCA). Hierarchical clustering was performed according to the Euclidean distance and complete linkage method. The data used for the clustering analysis were the relative abundance matrix, in which each row corresponds to a genus, and each column corresponds to a sample. Before clustering, data were filtered to eliminate genera with the sum of relative abundance across seven samples lower than 1%; remaining data were log_10-_transformed. A heatmap of the results of the clustering analysis and PCA were generated by using R/Bioconductor (www.R-project.org; www.bioconductor.org).

### Isolation and Enumeration of LAB

Mung beans collected from the various processing stages were washed with 9-fold volume of phosphate buffered saline. After vigorous vortexing, serial 10-fold dilutions were performed, and 0.1-mL aliquots of the appropriate dilutions were plated onto acidic MRS agar plates (pH 5.4; Diagnostic Systems, MD, USA) supplemented with 0.001% sodium azide and cycloheximide for LAB analysis. The plates were incubated at 30°C for 3 days in an anaerobic chamber (Coy Laboratory Products, Grass Lake, MI, USA; atmosphere comprising 85∶5:10 N_2_:CO_2_:H_2_). Colonies with distinct morphologies (that is, color, shape, and size) were selected and purified by streaking at least twice on MRS (pH 6.8) agar plates. The isolates were stored at –80°C in Nutrient Broth (Diagnostic Systems) containing 10% dimethyl sulfoxide.

### DNA Extraction

LAB isolates were cultured in MRS broth (pH 6.8) at 30°C for 1 to 2 days in an anaerobic chamber (Coy Laboratory Products) and harvested by centrifugation at 14,000×*g* for 3 min. For the genomic DNA extraction, the solid samples collected at the different processing stages were ground into powder, and diluted with 9-fold sterile saline (0.85% NaCl). After vigorous vortexing, 0.2-ml aliquots were used, as well as the liquid samples. The genomic DNA of LAB isolates or the total DNA of these samples was extracted as previously described [11]. Extracted DNA was dissolved in TE buffer (10 mM Tris-HCl, 1 mM EDTA, pH 8.0) for further analyses. To obtain total DNA, mung beans were suspended with 9-fold volume of phosphate buffered saline; total DNA was extracted from 200-µL aliquots of the suspension.

### RAPD Fingerprinting

RAPD-PCR analysis using random primers A (5′-CCGCAGCCAA-3′), B (5′-AACGCGCAAC-3′), and C (5′-GCGGAAATAG-3′) was performed as previously described [11]. The PCR products were separated by electrophoresis on a 1% agarose gel. Isolates with the same pattern were grouped, and a single representative strain from each group was chosen for subsequent gene analyses.

### Sequencing and Phylogenetic Analyses of 16S rRNA, pheS, and recA Genes

Approximately 1500 bases of each 16S rRNA gene sequence was amplified as previously described [11]. The primer sets and reaction conditions used for *pheS* gene amplification were those previously described [12], [13]. To identify strains in the *Lactobacillus plantarum* group (including *L. paraplantarum*, *L. pentosus*, and *L.* *plantarum*), *recA* sequences were used [14]. The PCR products were sequenced at the National Yang-Ming University Genome Research Centre. The methods for sequence assembly, similarity searches, and phylogenetic analyses have been described previously [15].

## Results

### Pyrosequencing-based 16S rRNA Profiling of Lu-doh-huang

High-throughput DNA analysis was applied to analyze changes in the total microbial composition during the manufacture of Lu-doh-huang. PCR yields from the UFM, S4–4M, and LDH samples were insufficient for further analysis due to the submarginal DNA-yield. A total of 71,792 sequence reads of the 16S rRNA gene were obtained from a single run by using barcoded pyrosequencing. We obtained a total of 71,623 high-quality reads, which had an average length of 340 bases and yielded approximately 24 Mb of sequence information. After barcode sorting, the read number of each sample varied from 6,622 to 14,473, with an average of 10,232 (Table 1). According to the Shannon–Weaver index, microbial diversity decreased as fermentation progressed during stage 1. However, the indices from S2–4M and S3–4M, corresponding to stages 2 and 3, respectively, were higher than those from stage 1. The microbial diversities of FSL and RSW were both higher than those of the samples that soaked in these liquids. The species evenness and rarefaction curves supported this finding (Fig. S1). We used rarefaction curves of reads at the 97% similarity level to estimate the richness of the bacterial communities. Except for the FSL and RSW samples, the OTUs detected were sufficient to represent the microbial composition present. In FSL, the numbers of genera and species detected were as many as 162 and 291, whereas RSW yielded 116 genera and 189 species. However, the sequence reads of these two samples were not sufficient to describe the entire microbial community, given that neither of the rarefaction curves reached a plateau.

**Table 1:** Pyrosequencing-based data from Lu-doh-huang samples.

	Sample	Total no. of reads	No. of high-quality reads	Average length(nt) of read	OTU	Shannon-Weaver index	Chao1	Evenness	No. of phyla	No. of genera	Total no.of species	No. of LAB species
Stage 1	S1–2W	11568	11560	330	124	2.73	149.59	0.57	3	49	84	21
	S1–1M	14486	14473	343	125	2.65	166.63	0.55	4	54	102	41
	S1–4M	7949	7937	348	61	2.09	71.91	0.51	4	39	66	26
Stage 2	FSL	10066	10052	336	485	4.13	616.47	0.67	8	162	291	36
	S2–4M	6626	6622	352	82	2.39	110.11	0.54	5	32	64	37
Stage 3	RSW	8999	8890	329	453	3.70	664.41	0.61	6	116	189	9
	S3–4M	12098	12089	339	213	2.45	288.30	0.46	6	79	170	63

Abbreviations: OTU, operational taxonomic unit. Chao1,

Shannon and evenness were calculated with Mothur at the 3% distance level.

### Bacterial Community Structures of Lu-doh-huang Samples

After classification, some sequences belonged to kingdom Plantae; phylum *Magnoliphyta* or *Prasinophyta*; genus *Agrostis*, *Glycine*, *Nephroselmis*, or *Nymphaea*. We concluded that chloroplast 16S rRNA genes that originated from the herbal medicines used during the production process or from the mung beans themselves were the source of these ‘outlier’ sequences, which were deleted from further analysis. Nine phyla (*Actinobacteria*, *Bacteroidetes*, *Chlorobi*, *Firmicutes*, *Proteobacteria*, *Synergistetes*, *Tenericutes*, *Thermotogae*, and *Verrucomicrobia*) were detected among all samples. Of these, phyla *Firmicutes*, *Proteobacteria,* and *Bacteroidetes* predominated. Sample FSL had the highest diversity, with 8 phyla, 162 genera, and 291 species of bacteria detected (Table 1).

**Figure 3:**
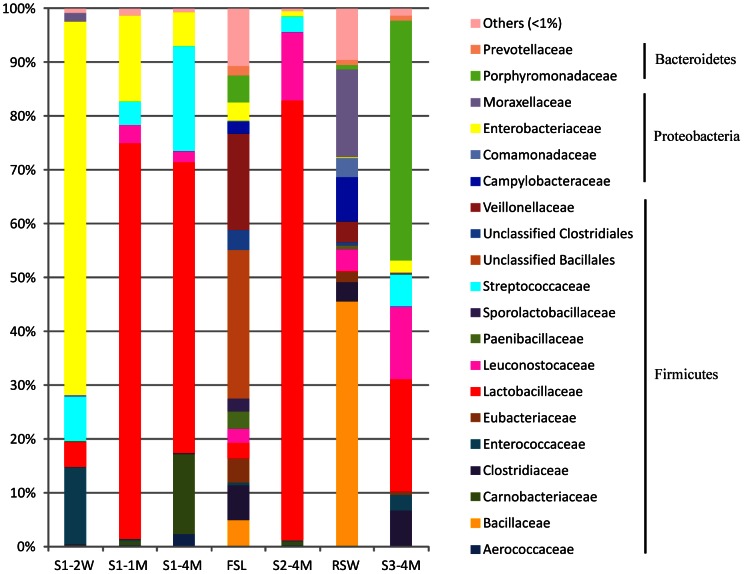
Bacterial community diversity during the fermentation of Lu-doh-huang. The relative abundance of bacterial 16S rRNA genes was estimated through classification at the family level.

Figure 3 presents the relative abundance of microbial components at family level in the Lu-doh-huang samples. In the earliest sample during fermentation (S1–2W), phylum *Proteobacteria* accounted for 71.9% of the bacterial community, in which family *Enterobacteriaceae* (69.3%) was predominant. The contribution of phylum *Firmicutes* increased as fermentation progressed during stage 1. In S1–1M and S1–4M, bacteria belonging to *Firmicutes* substituted for *Proteobacteria* and accounted for 82.8% and 93.1%, respectively, of the total population. In addition, the dominant bacteria shifted to *Lactobacillaceae* (73.5% and 53.9%, respectively) in these 2 samples. In S2–4M, phylum *Firmicutes* remained predominant (98.8%), in which the contribution from *Lactobacillaceae* increased to 81.6%. The bacterial composition of S3–4M was distinct, in that phylum *Firmicutes* (51.8%) decreased dramatically and was replaced by phylum *Bacteroidetes* (45.5%). The predominant family in S3–4M was *Porphyromonadaceae* (44.4%). The FSL was dominated by *Firmicutes* (82.1%), in which no particular bacteria was dominant and unclassified *Bacillales* (27.5%) and family *Veillonellaceae* (18.0%) were slightly more abundant than other families. In the RSW sample, phylum *Firmicutes* (62.7%) was dominant, followed by *Proteobacteria* (33.9%); the main family was *Bacillaceae* (45.5%).

Because the family *Lactobacilliaceae* (to which LAB belong) was the major bacteria, especially in samples collected within the bamboo sections, we performed a detailed analysis of these organisms at the species level (Table 2). A total of 264 genera and 586 species were obtained from all samples, among which 20 genera and 112 species were LAB and included *Aerococcus* (1 species), *Atopostipes* (1 species), *Bifidobacterium* (3 species), *Carnobacterium* (2 species), *Desemzia* (1 species), *Enterococcus* (14 species), *Facklamia* (3 species), *Globicatella* (1 species), *Ignavigranum* (1 species), *Lactobacillus* (54 species), *Lacticigenium* (1 species), *Lactococcus* (2 species), *Leuconostoc* (6 species), *Paralactobacillus* (1 species), *Pediococcus* (8 species), *Streptococcus* (2 species), *Tetragenococcus* (1 species), *Trichococcus* (1 species), *Vagococcus* (3 species), and *Weissella* (6 species). LAB abundances were 27.4%, 82.6%, 92.8%, 98.3%, and 43.2% in S1–2W, S1–1M, S1–4M, S2–4M, and S3–4M, respectively. The dominant LAB in these samples were *Enterococcus casselifavus* (11.7%) and *L. lactis* (8.2%) initially; these were replaced gradually by *Lactobacillus cacaonum* (33.2%) and *L. pentosus* (24.3%). Because *L. plantarum* and *L. pentosus* cannot be discriminated by use of their 16S rRNA gene sequences, *L. plantarum* should also contain according to culturing result. The contribution of these two bacteria increased as fermentation proceeded, reaching a maximum of 36.4% in S2–4M. *L. brevis* similarly increased over time, from 0.1% (S1–2W) to 30.7% (S2–4M). In S3–4M, the combined abundance of all LAB decreased to less than 10%. The abundance of LAB was only 6.0% in FSL and 4.4% in RSW. For samples in which family *Lactobacilliaceae* was not the main component, the dominant species were *Pragia fontium* (35.4%) in S1–2W, *Exiguobacterium mexicanum* (27.4%) in FSL, *Gracilibacillus boraciitolerans* (37.3%) in RSW, and *B. virosa* (40.4%) in S3–4M.

**Table 2:** Represented species composition of Lu-doh-huang.

	Species name	Stage 1			Stage 2		Stage 3	
		S1–2W	S1–1M	S1–4M	FSL	S2–4M	RSW	S3–4M
LAB	*Enterococcus casseliflavus*	11.7	0.2	–	0.1	–	–	<0.1
	*Enterococcus hirae*	–	<0.1	–	–	<0.1	–	1.5
	*Enterococcus mundtii*	1.0	–	–	–	–	–	–
	*Ignavigranum ruoffiae*	<0.1	0.1	2.2	–	0.2	–	<0.1
	*Lactobacillus brevis*	0.1	4.8	16.3	–	30.7	–	7.9
	*Lactobacillus cacaonum*	2.2	33.2	1.6	<0.1	0.2	–	0.1
	*Lactobacillus collinoides*	–	<0.1	–	–	11.2	–	0.3
	*Lactobacillus coryniformis*	–	–	–	<0.1	–	–	1.8
	*Lactobacillus hordei*	0.4	4.9	0.2	–	–	–	<0.1
	*Lactobacillus kimchii*	–	4.4	–	–	0.6	–	–
	*Lactobacillus mali*	0.0	0.3	3.2	–	0.1	–	<0.1
	*Lactobacillus pentosus*	1.9	24.3	31.8	0.1	36.4	–	6.7
	*Lactococcus lactis*	8.2	4.4	19.5	<0.1	2.7	–	5.9
	*Leuconostoc kimchii*	0.1	2.1	–	–	–	–	–
	*Leuconostoc mesenteroides*	<0.1	0.9	1.6	–	2.4	–	2.0
	*Pediococcus acidilactici*	–	–	–	–	–	–	1.5
	*Weissella fabaria*	–	–	–	2.3	–	4.0	8.7
	*Weissella hellenica*	<0.1	<0.1	<0.1	0.1	10.0	–	2.2
	Other LAB^a^	2.4	4.6	16.7	3.6	14.9	0.5	4.9
Other bacteria	*Acinetobacter baumannii*	–	–	<0.1	–	–	10.3	–
	*Anaerovibrio lipolyticus*	–	<0.1	–	6.6	–	1.4	0.1
	*Butyricimonas virosa*	<0.1	<0.1	–	2.2	<0.1	0.6	40.4
	*Enterobacter asburiae*	12.2	0.4	0.8	<0.1	–	–	0.1
	*Exiguobacterium mexicanum*	–	–	–	27.4	–	0.1	<0.1
	*Gracilibacillus boraciitolerans*	–	–	–	<0.1	–	37.3	–
	*Klebsiella pneumoniae*	9.9	1.6	0.1	0.1	<0.1	–	<0.1
	*Pragia fontium*	35.4	5.0	0.1	0.1	–	–	0.1
	Other bacteria^b^	15.1	12.4	7.2	60.1	1.7	48.0	16.4

a Sum of LAB for which relative abundance was less than 1% each.

b Sum of all other bacteria for which relative abundance was less than 5% each.

**Figure 4:**
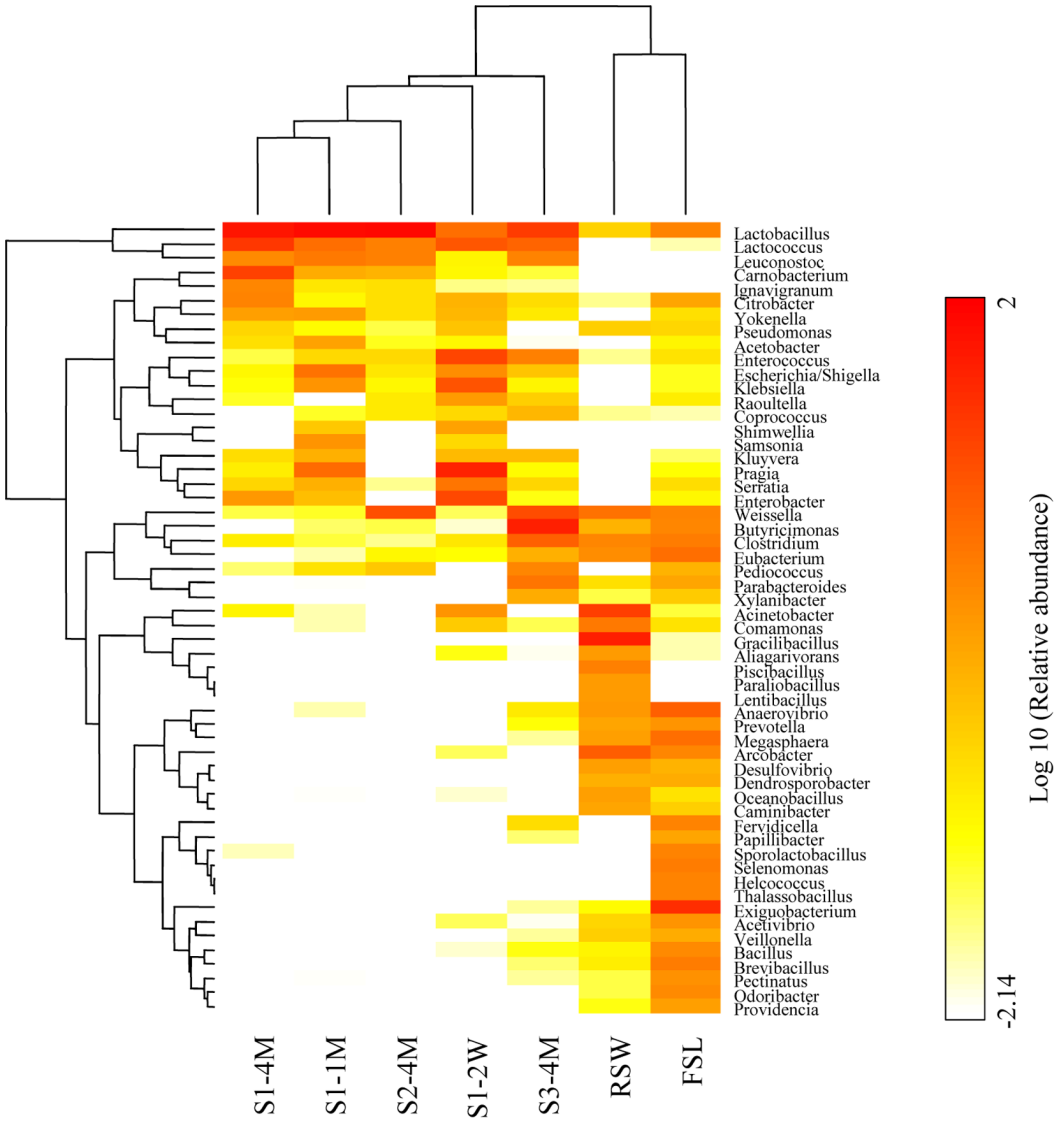
Phylogenetic tree and representative reads from the pyrosequencing analysis that only organisms with the sum of at least 1% relative abundance in all samples were included. Each column represents 1 sampling point; rows indicate different genera. The color intensity of the panel is proportional to the abundance of Log_10_ OTU.

**Figure 5:**
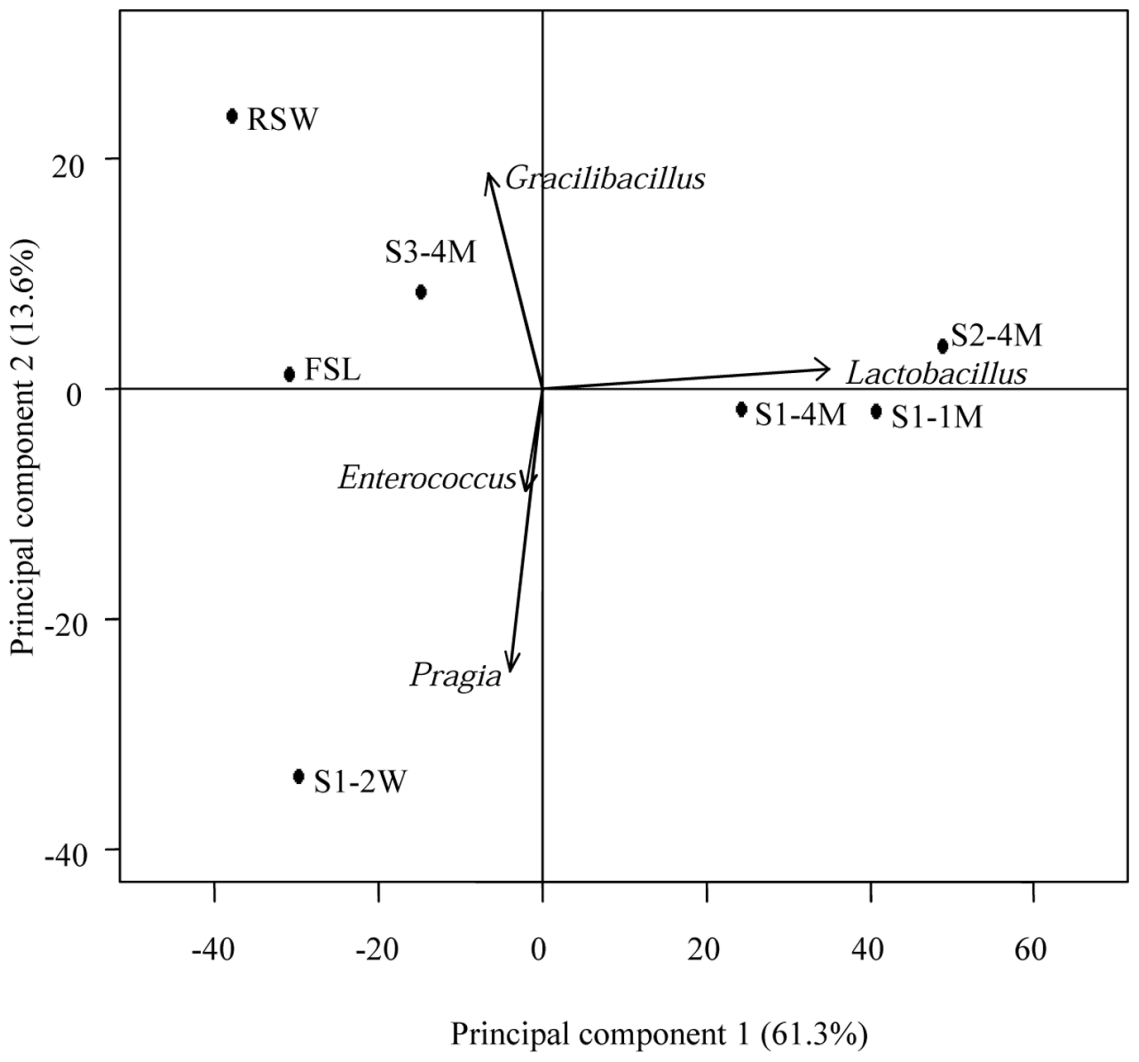
Principal component analysis plot derived from the relative abundance of bacterial communities at the genus level during Lu-doh-huang fermentation. The directions of arrows indicate the relative loading during the first and second principal components.

For all genera detected, 48 genera occupied 84.8% to 99.2% of the total bacterial community which the minimum incidence of 1% in a genus in each sample. To analyze the bacterial community associated with Lu-doh-huang fermentation in detail, we performed hierarchical clustering by using the relative abundance of genera (Fig. 4). The microflora of the environmental samples, FSL and RSW, formed a cluster separate from those representing the bacterial communities in mung bean samples obtained from bamboo sections (that is, S1–2W, S1–1M, S1–4M, S2–4M, and S3–4M). The dominant OTUs inside the bamboo sections were clearly dissimilar to those of environmental samples. Samples S1–1M, S1–4M, and S2–4M were close to each other in the hierarchical clustering, and the genus *Lactobacillus* was the strongest influencing factor for clustering. The remaining samples (S1–2W, FSL, RSW, and S3–4M) were well separated from other clusters. In addition, we performed PCA to characterize the microbial community succession of Lu-doh-huang (Fig. 5). A projection of the first two principal components explained about 75% (61.3% and 13.6%) of the total variance, with strong separation by region.

### pH and Cell Counts of LAB of Lu-doh-huang Samples

The pH of the unfermented starting material (UFM), which contained mung beans and various Chinese medicines, was 5.4 (Table 3). After this mixture was stuffed into bamboo sections for 2 weeks (S1–2W), the pH increased slightly to 5.9. However, pH started to decrease gradually and 4 months later, it was 3.9 (S1–4W). During stages 2 and 3 (S2–4M and S3–4M), pH increased to approximately 5.0. The pH of the FSL and RSW samples was 5.5 and 6.9, respectively.

**Table 3:** The pH values and cell counts of lactic acid bacteria (LAB) in Lu-doh-huang.

	Stage 1				Stage 2		Stage 3		Stage 4	
	UFM	S1–2W	S1–1M	S1–4M	FSL	S2–4M	RSW	S3–4M	S4–4M	LDH
pH value	5.4	5.9	5.3	3.9	5.5	4.8	6.9	5.1	5.3	4.9
LAB	–a	3.5×10^8b^	4.9×10^7^	1.6×10^8^	1.1×10^4^	6.3×10^7^	1.4×10^4^	3.5×10^7^	–	–

a Not detected.

b No. of LAB cfu/g or cfu/mL.

The traditional culture method detected no LAB in the UFM, S4–4M, and LDH samples (Table 3). After anaerobic fermentation for 2 weeks inside bamboo sections, the cell counts of LAB increased to the level of 10^8^ cfu/g. This cell count remained stable throughout the manufacturing process, until the fermented mung beans were removed from the bamboo sections. However, sample S4–4M lacked LAB, which were isolated from FSL and RSW at a level of about 10^4^ cfu/mL.

### Identification and Distribution of LAB

LAB strains were then isolated from each sample according to their colony morphologies and analyzed by RAPD-PCR for grouping and 16S rRNA gene sequencing for identification. In total, 218 strains were isolated (7 from UFM, 35 from S1–2W, 16 from S1–1M, 24 from S1–4M, 16 from FSL, 72 from S2–4M, 16 from RSW, and 32 from S3–4M). No colonies grew on MSR agar streaked with the LDH sample. On the basis of the resulting RAPD profiles, 218 isolates were categorized into 82 different groups (5 from UFM, 10 from S1–2W, 9 from S1–1M, 4 from S1–4M, 9 from FSL, 25 from S2–4M, 9 from RSW, and 11 from S3–4M). A representative strain was chosen from each of the 82 groups for partial sequencing of the 16S rRNA gene (500 nucleotides containing the V1 to V3 regions) [16]; the sequences obtained were identified as belonging to 4 genera (*Enterococcus*, *Lactobacillus*, *Lactococcus,* and *Pediococcus*) and 24 species. Most of the isolates were *Lactobacillus*, comprising 19 different species. The strains from UFM were not LAB.

Sample S1–2W contained 6 different LAB species; *Enterococcus casseliflavus* and *Lactococcus lactis* were the dominant species, with cell counts as high as 10^8^ cfu/g (Table 4). As the fermentation proceeded, the number of LAB species decreased, and the main species shifted to *Lactobacillus brevis* and *L. plantarum* (S1–1M and S1–4M). After the bamboo sections had been opened to soak in the fermented soybean liquid for 4 months (S2–4M), the diversity of LAB had changed dramatically. The 10 LAB species obtained were present in similar numbers, and *L. plantarum* was the only species that remained from stage 1. None of the LAB species isolated from FSL was present in S2–4M. Similarly, except for *L. brevis*, none of the LAB in RSW was found in S3–4M. LAB cell counts increased slightly between stages 2 and 3, and *L. brevis* and *L. pentosus* predominated during both stages.

**Table 4:** Lactic acid bacteria (LAB) composition in the different stages of Lu-doh-huang.

LAB species	1st stage			2nd stage		3rd stage	
	S1–2W	S1–1M	S1–4M	FSL	S2–4M	RSW	S3–4M
*Enterococcus casseliflavus*	8.18^a^	–	–	–	–	–	–
*Lactobacillus acidipiscis*	–	–	–	2.48	–	3.91	–
*Lactobacillus brevis*	6.27	6.60	7.79	–	–	2.00	7.23
*Lactobacillus buchneri*	–	–	–	3.88	–	3.45	–
*Lactobacillus casei*	–	–	–	–	5.04	–	–
*Lactobacillus collinoides*	–	–	–	–	5.51	–	–
*Lactobacillus concavus*	–	–	–	–	–	2.70	–
*Lactobacillus farciminis*	–	–	–	–	5.88	–	–
*Lactobacillus hilgardii*	–	–	–	3.13	–	–	–
*Lactobacillus kimchii*	–	–	–	–	–	–	–
*Lactobacillus paralimentarius*	–	6.78	–	–	–	–	–
*Lactobacillus pentosus*	–	–	–	–	–	–	7.09
*Lactobacillus perolens*	–	–	–	–	–	–	–
*Lactobacillus plantarum*	6.85	7.59	7.89	–	–	–	–
*Lactobacillus similis*	–	–	–	2.93	–	–	–
*Lactobacillus vaccinostercus*	–	–	–	–	–	–	–
*Lactobacillus sp.* A	6.56	–	–	–	–	–	–
*Lactobacillus sp.* B	7.13	–	–	–	–	–	–
*Lactobacillus sp.* C	–	–	–	–	–	–	5.56
*Lactobacillus sp.* D	–	–	–	–	–	–	6.47
*Lactococcus lactis*	8.23	–	–	–	–	–	–
*Pediococcus acidilactici*	–	–	–	–	–	–	–
*Pediococcus cellicola*	–	–	–	–	6.08	–	–
*Pediococcus parvulus*	–	–	–	–	6.08	–	–
							–
							–

a cfu/g (ml). Cell counts are given as Log10 cfu/g or cfu/mL, depending on the sample (mung beans or soaking liquid).

Among the *Lactobacillus* species identified, 6 representative strains isolated from S1–2W and S3–4W could not be identified conclusively by using the full-length 16S rRNA gene sequences because these sequences were more than 99% or less than 97% similar to those of known species in the databases. Therefore, we compared *pheS* gene sequences of these 6 strains. Because the *pheS* sequence identities of these strains were all lower than 90% with recognized species (data not shown), these isolates were tentatively identified as *Lactobacillus* sp. A, B, C, and D.

## Discussion

In this study, we used pyrosequencing of tagged 16S rRNA gene amplicons and the traditional culture method to reveal changes in microbioflora of Lu-doh-huang during its production. Lu-doh-huang is a unique regional and alternative medicine that is especially popular in southern Taiwan. To produce Lu-doh-huang, mung beans are mixed with other Chinese herbal medicines, fermented anaerobically, soaked in liquid herbal medicines repeatedly, and then dried completely. A similar product is Semen Sojae Praeparatum in mainland China. The processing of Lu-doh-huang and Semen Sojae Praeparatum is comparable: both combine a type of bean (mung beans and black beans, respectively) with other herbal medicines and undergo natural fermentation. A key difference between these products is the type of fermentation used in their manufacture, that is, Lu-doh-huang undergoes strictly anaerobic fermentation (inside bamboo sections), whereas the production of Semen Sojae Praeparatum involves aerobic fermentation. However, the microflora of Semen Sojae Praeparatum has not been analyzed extensively [17].

Natural fermentation is a common production method for Chinese herbal medicines that has been used for almost 2,000 years. Many medicines produced through natural fermentation are still popular, including Massa Medicata Fermentata, medicated leaven, Semen Sojae Praeparatum, and Chinese gall leaven [18]. Microbial enzymes present during fermentation are thought to convert or detoxify bioactive compounds in the medicinal herbs to enhance the health benefits of the final product [18], [19]. For example, gastrointestinal bacteria transform bioactive ingredients of herbal medicines so that they become easily absorbed or pharmacologically active [20]–[24]. *Aspergillus* spp. present in fermented *Radix astragalus* significantly increased the phenolic content and antioxidant activity of the product compared with those of the unfermented form [25]. *Bacillus subtilis* natto and fermented *Radix astragali* stimulated hyaluronic acid production and increased the mRNA expression of hyaluronan synthase 3 and hyaluronan synthase 2 in HaCaT cells and human fibroblasts, respectively [26]. Semen Sojae Praeparatum had greater quantities of isoflavone glucosides and aglycones, which show extensive pharmacologic activities, than did natural soy materials [27]. In our study, LAB were the major bacterial components of Lu-doh-huang, especially in samples S1–1M, S1–4M, and S2–4M, which were obtained while the mung beans were fermenting inside the bamboo sections. Fermentation by LAB reportedly increased the pharmacologic effects of several herbal medicines and detoxified various dietary carcinogens [28]–[33]. Therefore, we speculate that the main purpose of the unique production process of Lu-doh-huang is to enable microbial enzymes secreted during fermentation to biotransform various components of the product.

Genus *Lactobacillus* was the most frequent and predominant LAB detected by both pyrosequencing and culturing. Pyrosequencing revealed greater diversity in *Lactobacillus* (54 species) than did culturing (19 species). However, culturing–but not pyrosequencing–detected *L. acidipiscis* (at 10^3.91 ^cfu/g in RSW), *L. brevis* (at 10^2.00 ^cfu/g in RSW), *L. casei* (at 10^5.04 ^cfu/g in S2–4M), *L. concavus* (at 10^2.70 ^cfu/g in RSW), *L. farciminis* (at 10^5.88 ^cfu/g in S2–4M), *L. hilgardii* (at 10^3.13 ^cfu/g in FSL), *L. plantarum* (at 10^6.85^, 10^7.59^, 10^7.89^, and 10^5.04 ^cfu/g in S1–2W, S1–1M, S1–4M, and S2–4M, respectively), and *L. similis* (at 10^2.93 ^cfu/g in FSL). The dissimilarity in the results obtained by using culturing and pyrosequencing may reflect the fact that the V1–V2 region of 16S rRNA does not sufficiently discriminate some species to enable their precise classification. For example, members of the *L. plantarum* group–*L. fabifermentans*, *L. pentosus*, *L. paraplantarum*, and *L. plantarum*–showed high sequence similarity (97.2%–99.7%). In fact, these values generally were considerably higher than the threshold level typically recommended for species differentiation (97%) [34]. Therefore, additional genetic biomarkers were needed to accurately speciate members of the *L. plantarum* group that were identified by BLAST searching of the pyrosequencing reads [14], [35].

Additional data garnered through sequencing of *recA* and culturing indicated that the species *L. fabifermentans*, *L. paraplantarum*, and *L. pentosus* that were identified through pyrosequencing (data not shown) should be classified instead as *L. plantarum*, whereas *L. pentosus* in S2–4M actually comprises both *L. pentosus* and *L. plantarum* (data not shown). In contrast, *L. hilgardii* and *L. parakefiri* in the *L. buchneri* group; *L. farciminis* in the *L. farciminis* group, could be identified appropriately on the basis of the similarity values of the V1–V2 region of the 16S rRNA gene (which were lower than 97%) that were obtained by pyrosequencing (except in the pairs, *L. casei* and *L. paracasei* in the *L. casei* group, and *L. bobalius* and *L. paralimentarius* in the *L. farciminis* group), even though similarity values of the full-length 16S rRNA sequences were considerably higher (>97.3%) (data not shown).

Another possible reason for the dissimilarities between the results of culturing and pyrosequencing may be in the different detection limits of the two methods: 10^2 ^cfu/g (or cfu/mL, depending on the type of sample) for culturing compared with 10^5^–10^6 ^cfu/g (or cfu/mL) for pyrosequencing (estimated the concentration of isolated bacteria from the conversion of the abundance of pyrosequencing reads). The different limits of detection account for why *L. acidipiscis, L. brevis, L. concavus* and *L. similis* were not identified through pyrosequencing of FSL or RSW, whereas these organisms were isolated in concentrations ranging from 10^2^ to 10^3.91 ^cfu/mL through culturing. In contrast, *L. cacaonum* was detected as the predominant species (33.2%) in S1–1M by pyrosequencing, but culturing failed to yield this organism. Perhaps only dead (and therefore unculturable) cells were present at this stage: DNA-based methods such as pyrosequencing do not distinguish viable from dead cells.

The results of heatmap and PCA plot indicated that the microbial communities of S1–2W and S3–4M were markedly different from those of S1–1M, S1–4M, and S2–4M. Early during fermentation of the mung beans inside bamboo sections, air was consumed gradually to form an anaerobic environment. In addition, various compounds derived from the herbal medicines that were mixed with the mung beans have antimicrobial activity [36]–[38]. These features caused the microbial composition during stage 1 to be more unstable than those at later sampling points. By the end of production, facultative or obligate anaerobes, such as *Lactobacillaceae*, *P. fontium* and *B. virosa,* had become predominant. The predominant microflora inside the bamboo sections began as phylum *Proteobacteria* (S1–2W, 71.9%) and then gradually shifted to phylum *Firmicutes* (28.1%, 82.8%, 93.1%, and 98.8% in S1–2W, S1–1M, S1–4M, and S2–4M, respectively). However, in S3–4M, the microbial composition was distinct in that phylum *Bacteroidetes* (45.5%) substituted *Firmicutes*. Given that the microbial composition of the environmental samples (FSL and RSW) differed markedly from those collected from inside the bamboo sections according to the pyrosequencing and culture results, bacteria seem to be unable to pass from the solutions, through the bamboo sections, and into the mung beans. Instead, perhaps the pH of or small molecules in the soaking solutions influence the bacterial succession inside the bamboo sections.

In conclusion, we used culturing and high-throughput pyrosequencing analyses to characterize the microbial community of the Lu-doh-huang fermentation ecosystem. Our work provides a detailed picture of the structures of the microbiomes of 7 samples obtained at different points during the manufacturing process. Results from pyrosequencing generally were consistent with the culture-dependent analysis and provided deeper insight into the entire prokaryotic microbial communities at the various time points. The details of relationship the microbes in Lu-doh-huang and its functions are still unclear. Further investigations are needed to shed light on the microbial dynamics, and the analysis of bioactive components would contribute to a better understanding of this Taiwanese alternative medicine.

## Supporting Information

Figure S1 **Rarefaction curves for each sample were calculated at 3% distance by using the RDP pipeline data of the 16S rRNA gene sequences.**
(TIF)
