# Supplementary figures and images for: Microbial Diversity Analysis of Fermented Mung Beans (Lu-Doh-Huang) by Using Pyrosequencing and Culture Methods
Source: PLoS One. 2013 May 20;8(5):e63816. doi: 10.1371/journal.pone.0063816 (PMC3659079; doi:10.1371/journal.pone.0063816)

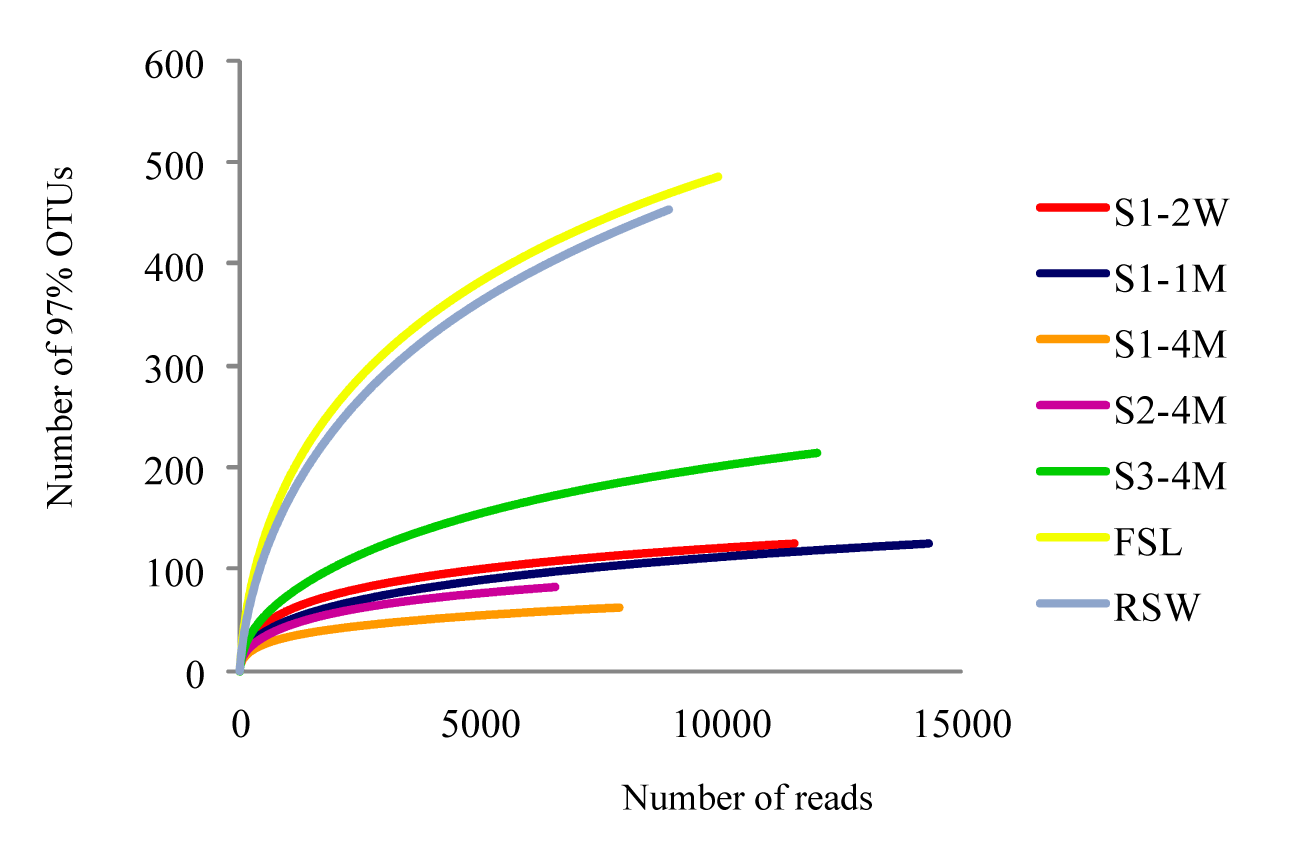

Supplement: Figure S1 — Rarefaction curves for each sample were calculated at 3% distance by using the RDP pipeline data of the 16S rRNA gene sequences. (TIF) [file pone.0063816.s001.tif]
